# A greater birthweight increases the risk of acute leukemias in Mexican children—experience from the Mexican Interinstitutional Group for the Identification of the Causes of Childhood Leukemia (MIGICCL)

**DOI:** 10.1002/cam4.1414

**Published:** 2018-03-13

**Authors:** Elva Jiménez‐Hernández, Arturo Fajardo‐Gutiérrez, Juan Carlos Núñez‐Enriquez, Jorge Alfonso Martín‐Trejo, Laura Eugenia Espinoza‐Hernández, Janet Flores‐Lujano, José Arellano‐Galindo, Aurora Medina‐Sanson, Rogelio Paredes‐Aguilera, Laura Elizabeth Merino‐Pasaye, Martha Margarita Velázquez‐Aviña, José Refugio Torres‐Nava, Rosa Martha Espinosa‐Elizondo, Raquel Amador‐Sánchez, Juan José Dosta‐Herrera, Javier Anastacio Mondragón‐García, Heriberto Valdés‐Guzmán, Laura Mejía‐Pérez, Gilberto Espinoza‐Anrubio, María Minerva Paz‐Bribiesca, Perla Salcedo‐Lozada, Rodolfo Ángel Landa‐García, Rosario Ramírez‐Colorado, Luis Hernández‐Mora, María Luisa Pérez‐Saldivar, Marlene Santamaría‐Ascencio, Anselmo López‐Loyola, Arturo Hermilo Godoy‐Esquivel, Luis Ramiro García‐López, Alison Ireri Anguiano‐Ávalos, Karina Mora‐Rico, Alejandro Castañeda‐Echevarría, Roberto Rodríguez‐Jiménez, José Alberto Cibrian‐Cruz, Karina Anastacia Solís‐Labastida, Rocío Cárdenas‐Cardos, Armando Martínez‐Avalos, Luz Victoria Flores‐Villegas, José Gabriel Peñaloza‐González, Ana Itamar González‐Ávila, Martha Beatriz Altamirano‐García, Norma López‐Santiago, Martin Sánchez‐Ruiz, Roberto Rivera‐Luna, Luis Rodolfo Rodríguez‐Villalobos, Francisco Hernández‐Pérez, Jaime Ángel Olvera‐Durán, Luis Rey García‐Cortés, Minerva Mata‐Rocha, Omar Alejandro Sepúlveda‐Robles, Cesar Raúl González‐Bonilla, Vilma Carolina Bekker‐Méndez, Silvia Jiménez‐Morales, Haydee Rosas‐Vargas, Juan Manuel Mejía‐Aranguré

**Affiliations:** ^1^ Health Research Coordination Instituto Mexicano del Seguro Social (IMSS) Mexico City Mexico; ^2^ Pediatric Hematology Services Hospital General “Gaudencio González Garza” CMN “La Raza” IMSS Mexico City Mexico; ^3^ Medical Research Unit in Clinical Epidemiology UMAE Hospital de Pediatría CMN “Siglo XXI” IMSS Mexico City Mexico; ^4^ Hematology Services UMAE Hospital de Pediatría CMN “Siglo XXI” IMSS Mexico City Mexico; ^5^ Children's Hospital of Mexico, Federico Gómez Secretaria de Salud (SS) Mexico City Mexico; ^6^ Hematology Services Instituto Nacional de Pediatría (INP), SS Mexico City Mexico; ^7^ Pediatric Hematology Services CMN “20 de Noviembre” Instituto de Seguridad Social al Servicio de los Trabajadores del Estado (ISSSTE) Mexico City Mexico; ^8^ Onco‐Pediatrics Service Hospital Juárez de México, SS Mexico City Mexico; ^9^ Oncology Services Hospital Pediátrico “Moctezuma” Secretaría de Salud de la Ciudad de México (SSCDMX) Mexico City Mexico; ^10^ Pediatric Hematology Service Hospital General de México, SSa Mexico City Mexico; ^11^ Pediatric Hematology Service Hospital General Regional (HGR) No. 1 “Dr. Carlos Mac Gregor Sánchez Navarro” IMSS Mexico City Mexico; ^12^ Pediatric Surgery Service Hospital General “Gaudencio González Garza” CMN “La Raza”, IMSS Mexico City Mexico; ^13^ Pediatric Surgery Service HGR No. 1 “Dr. Carlos Mac Gregor Sánchez Navarro” IMSS Mexico City Mexico; ^14^ Pediatric Hospital of Iztacalco SSCDMX Mexico City Mexico; ^15^ Pediatric Hospital of Iztapalapa SSCDMX Mexico City Mexico; ^16^ Servicio de Pediatría Hospital General Zona (HGZ) No. 8 “Dr. Gilberto Flores Izquierdo” IMSS Mexico City Mexico; ^17^ Pediatric Services Hospital Juárez del Centro, SS Mexico City Mexico; ^18^ General Hospital of Ecatepec “Las Américas” Instituto de Salud del Estado de México (ISEM) Mexico City Mexico; ^19^ General Hospital “Dr. Manuel Gea González” SS Mexico City Mexico; ^20^ Pediatric Hospital “La Villa” SSCDMX Mexico City Mexico; ^21^ Pediatric Hospital “San Juan de Aragón” SSCDMX Mexico City Mexico; ^22^ Pediatric Services HGR No. 72 “Lic. Vicente Santos Guajardo”, IMSS Mexico City Mexico; ^23^ Pediatric Surgery Service, HGZ No. 32 IMSS Mexico City Mexico; ^24^ Pediatric Surgery Service Hospital Pediátrico de Moctezuma SSCDMX Mexico City Mexico; ^25^ Pediatric Services Hospital Pediátrico de Tacubaya SSCDMX Mexico City Mexico; ^26^ Pediatric ER, HGZ No. 47 IMSS Mexico City Mexico; ^27^ Pediatric Surgery Service Hospital Regional “1° Octubre” ISSSTE Mexico City Mexico; ^28^ HGR No. 25 IMSS Mexico City Mexico; ^29^ Pediatric Services Hospital General de Zona con Medicina Familiar (HGZMF) No. 29 IMSS Mexico City Mexico; ^30^ Pediatric Surgery Service, HGZ No. 27 IMSS Mexico City Mexico; ^31^ Oncology Services INP, SSa Mexico City Mexico; ^32^ Delegación Regional Estado de México Oriente IMSS Mexico City Mexico; ^33^ Molecular Biology Laboratory UMAE, Hospital de Pediatría CMN “Siglo XXI” IMSS Mexico City Mexico; ^34^ Laboratory of the Coordination of Epidemiological Surveillance and Support in Contingencies Unidad de Investigación Médica en Inmunología e Infectología Hospital de Infectología “Dr. Daniel Méndez Hernández” CMN “La Raza” IMSS Mexico City Mexico; ^35^ Medical Research Unit in Immunology and Infectology Hospital de Infectología “Dr. Daniel Méndez Hernández” CMN “La Raza” IMSS Mexico City Mexico; ^36^ Cancer Genomics Laboratory Instituto Nacional de Medicina Genómica Mexico City Mexico; ^37^ Medical Research Unit in Human Genetics UMAE, Hospital de Pediatría CMN “Siglo XXI” IMSS Mexico City Mexico

**Keywords:** Birthweight, children, epidemiology, leukemia, risk factors

## Abstract

In Mexico, due to the high rates of diabetes, overweight, and obesity, there has also been noted an increased newborn weight, which may be contributing to the elevated incidence rate of childhood acute leukemia (AL). We conducted a case–control study in public hospitals of Mexico City aimed to know whether a greater weight at birth is associated with a higher risk of developing leukemia. We included incident cases with acute lymphoblastic leukemia (ALL) and acute myeloid leukemia (AML) diagnosed between 2010 and 2015. Controls were frequency‐matched to the cases by age, sex, and health institution. Logistic regression analysis was performed adjusting risks by child's sex, overcrowding index, birth order, and mother's age at the time of pregnancy. Adjusted odds ratios (aORs) and 95% confidence intervals were calculated. A total of 1455 cases and 1455 controls were included. An evident association between ALL and child's birthweight ≥2500 g was found (aOR 2.06; 95% CI: 1.59, 2.66) and also, in those with birthweight ≥3500 g (aOR 1.19; 95% CI: 1.00, 1.41). In AML patients with birthweight ≥2500 g and ≥3500 g, an aOR of 1.77 (95% CI: 1.07, 2.94) and 1.42 (95% CI: 1.03–1.95) was observed, respectively. No association was noticed with either type of AL and a birthweight ≥4000 g. To sum up, we found a moderate association between not having a low birthweight and an increased risk of acute leukemias. Birthweight ≥3500 g was also a risk factor for both types of leukemia. This suggests that a greater birthweight may increase the risk of acute leukemias in Mexican children.

## Introduction

Acute leukemia (AL) is consistently the most common cancer in children, almost one‐third of all types before the age of 15 years 1, 2. The highest incidence rates have been reported in the Hispanic population residing in the United States, Costa Rica, and Mexico City 3, 4, 5, 6.

Great advances have been achieved in the last 20 years in the knowledge on the biology of leukemia 7, 8, 9, and there has also been an increase in long‐term survival through improvements in treatment regimens and supportive care 10, 11, 12. However, there is little known of its etiology, with the existence of different morphological subtypes, great heterogeneity in pathophysiology, clinical manifestations, variability in response to treatment and prognosis, all of these suggesting different etiologies 13, 14, 15. Epidemiological investigations have proposed many potential risk factors 16, 17, 18, 19, 20 although few have been confirmed, mainly for acute myeloid leukemia (AML) 21, 22, 23.

In recent years, evidence has emerged pointed out that the development of childhood acute lymphoblastic leukemia (ALL) and AML is initiated in utero, particularly, because cells carry certain chromosomal translocations specific for leukemic cells such as *t*(12:21), *t*(4:11), or *t*(8:21) present at the time of birth in those children later diagnosed with leukemia 24, 25, 26, 27, 28. This emphasizes the importance of prenatal exposure to leukemogenic factors in the unfolding of this disease.

Birthweight is influenced not only by genetics but also by the exposure to several intrauterine environment factors as well, and although it has remained poorly characterized, it is presumed to be associated with acute leukemia in children 29, 30, 31, 32. The results of a meta‐analysis of 18 case–control studies reported that birthweight >4000 g was associated with an increased risk of ALL [Odds ratio (OR) 1.26 (95% CI: 1.17–1.37] 33. More recent studies have consistently mentioned that an accelerated fetal growth rather than a high birthweight per se is determinant in childhood leukemia 34, 35. In a pooled analysis of 12 case–control studies documented by the Childhood Leukemia International Consortium (CLIC), it is noted that those children with an appropriate weight for gestational age had a modest increase in ALL risk [OR 1.16 (95% CI: 1.09–1.24], evidencing this, in the absence of high weight at birth 36.

Mexico is a country with high rates of diabetes, overweight, and obesity, also affecting women at reproductive age 37, 38. These could potentially imply the presence of fetal complications, likewise fetal macrosomia and/or being larger for gestational age, both related to an accelerated fetal growth 39, 40. If a greater birthweight is a risk factor for developing childhood AL, this could be contributing to the increased incidence rates reported in our population. Therefore, the objective of this study was to assess this association.

## Materials and Methods

A case–control study was conducted. Cases were children with ALL or AML diagnosed between 2010 and 2015, and controls were children without AL and frequency‐matched to the cases by age, sex, and health institution. The project was approved in all participant institutions with the number R‐2008‐785‐063 of the National Commission of Scientific Research.

### Cases

In Mexico City, there are both private and public hospitals attending children with leukemia. Private hospitals attend approximately 5% of all children with leukemia, and the remaining 95% of cases are treated in one of the nine participant public hospitals, affiliated to one of the following Institutions of Health: (1) Secretaría de Salud (SS): Hospital General de México, Hospital Juárez de México, Hospital Infantil de México “Federico Gómez”, and the Instituto Nacional de Pediatría; (2) Instituto Mexicano del Seguro Social (IMSS): Unidad Médica de Alta Especialidad (UMAE) Centro Médico Nacional “La Raza,” UMAE Hospital de Pediatría del Centro Médico Nacional Siglo XXI “Dr. Silvestre Frenk Freund”, and the Hospital General Regional No. 1 Carlos McGregor Sánchez Navarro; (3) Instituto de Seguridad y Servicios Sociales de los Trabajadores del Estado (ISSSTE): Hospital 20 de Noviembre and (4) Secretaría de Salud de la Ciudad de México: Hospital Pediátrico Moctezuma.

All cases were diagnosed according to child's clinical features, bone marrow aspiration, for morphological classification according to FAB criteria, cytochemical staining, and immunophenotype. During the period of the study, 1748 cases were identified; however, we could not obtain matching controls for all these patients, and therefore, not all cases were included in the present analysis. Characteristics of leukemia cases, included or not included, are displayed as supplementary information (Table S1).

### Controls

Controls were recruited from second‐level hospitals, from where cases were referred to third‐level care hospitals for diagnosis confirmation. Controls were admitted to those hospitals for fractures, surgical diseases (tonsillectomy, adenotonsillectomy, hernioplasty, circumcision, orchidopexy, phimosis, and varicocele), and nonsurgical diseases (reflux, gastrointestinal infection, salmonellosis, dehydration, asthmatic crisis, pneumonia, epileptic seizures, optic infection, burns). One control per case was matched. The parents of each child were informed of what the study consisted of and consent was obtained to participate in the study. Controls (*n* = 1455) were interviewed during the next periods: 2002 (*n* = 2; 0.1%), 2004 (*n* = 52; 3.6%), 2005 (*n* = 113; 7.8%), 2006 (*n* = 22; 1.5%), 2007 (*n* = 148; 10.2%), 2010 (*n* = 200; 13.8%), 2011 (*n* = 359; 24.7%), 2012 (*n* = 418; 28.7%), 2013 (*n* = 30; 2.1%), 2014 (*n* = 13; 0.9%), and during 2015 (*n* = 98; 6.7%).

### Data collection

Interviews were carried out by trained personnel using the same questionnaire for cases and controls which is a previously standardized one adapted from the questionnaire module of the National Cancer Institute 41. We obtained demographic information such as birthweight, gender, parents’ age at pregnancy, birth order, whether the child was breastfed, and education level of the mother. The cases parents’ interviews were performed within the first 2 months after diagnosis.

Diagnostic data were obtained directly from patient's clinical record. Birthweight was grouped as in other studies, in the following categories: <2500 g (low birthweight), ≥2500 to 3499 g (appropriate birthweight, using this category as reference), 3500–4499.99 g (high birthweight) and ≥4500 g (very high birthweight) 34, 35, 42, 43. In addition, we categorized birthweight as the following dichotomous variables <2500 g and ≥2500 g, <3500 g and ≥3500 g and <4000 g and ≥4000 g 44.

### Statistical analysis

Descriptive statistics were performed, and frequency measurements and percentages were calculated. Categorical variables were compared between cases and controls using chi‐square test and/or Fisher's exact test as appropriate. Afterward, a logistic regression analysis was conducted adjusting by child's sex, overcrowding index, birth order, and mother's age at the time of pregnancy. Adjusted odds ratios (aOR) were calculated with 95% confidence intervals (95% CI). The software used was SPSS IBM (Statistical Package for the Social Sciences, Inc., Version 20, Chicago, IL).

## Results

A total of 2910 children, 1455 cases, and 1455 controls were analyzed. The median age of the population at diagnosis (cases) and/or at the time of interview (controls) was 6.1 years (range: 0–17.6 years). Tables 1 and 2 display the distribution of sex, birthweight, age at diagnosis, and other characteristics between cases and controls. Nonfirstborn in the controls was 54%, whereas in the cases was 59.4%. Breastfeeding frequency was slightly higher in cases (91.4%) than in controls (87.4%). Regarding mothers’ age at the time of pregnancy in both, cases and controls, most were between 20 and 34.99 years.

**Table 1:** Birthweight of cases and controls and other characteristics

Study variables	Controls *n* = 1455 *n* (%)	AL cases *n* = 1455 *n* (%)	OR (95% CI)	ALL *n* = 1253 *n* (%)	OR (95% CI)	AML *n* = 202 *n* (%)	OR (95% CI)
Child's sex							
Male	837 (57.5)	778 (53.5)	0.84 (0.73–0.98)	668 (53.3)	0.84 (0.72–0.98)	110 (54.5)	0.88 (0.65–1.18)
Female	618 (42.5)	677 (46.5)	–	585 (46.7)	–	92 (45.5)	–
Birthweight (grams)							
≥2500–3499	874 (60.1)	913 (62.7)	–	794 (63.4)	–	119 (58.9)	–
<2500	211 (14.5)	114 (7.8)	0.51 (0.40–0.66)	96 (7.7)	0.50 (0.38–0.64)	18 (8.9)	0.62 (0.37–1.05)
≥3500–4000	362 (24.9)	418 (28.7)	1.10 (0.93–1.30)	355 (28.3)	1.07 (0.90–1.28)	63 (31.2)	1.27 (0.92–1.77)
>4000	8 (0.5)	10 (0.7)	1.19 (0.47–3.04)	8 (0.6)	1.10 (0.41–2.94)	2 (1.0)	1.83 (0.38–8.74)
Birthweight (grams)							
<4000	1383 (95.1)	1378 (94.7)	–	1190 (95.0)	–	188 (93.1)	–
≥4000	72 (4.9)	77 (5.3)	1.07 (0.77–1.49)	63 (5.0)	1.01 (0.71–1.43)	14 (6.9)	1.43 (0.79–2.58)
Birthweight (grams)							
<3500	1085 (74.6)	1026 (70.5)	*–*	890 (71.0)	*–*	136 (67.3)	*–*
≥3500	370 (25.4)	429 (29.5)	1.22 (1.04–1.44)	363 (29.0)	1.19 (1.01–1.41)	66 (32.7)	1.42 (1.03–1.95)
Birthweight (grams)							
<2500	211 (14.5)	114 (7.8)	–	96 (7.7)	–	18 (8.9)	–
≥2500	1244 (85.5)	1341 (92.2)	1.99 (1.56–2.53)	1157 (92.3)	2.04 (1.58–2.63)	184 (91.1)	1.73 (1.04–2.87)

AL, acute leukemias; ALL, acute lymphoblastic leukemia; AML, acute myeloid leukemia.

**Table 2:** Characteristics of cases and controls

*N* = 2910	*n* = 1455 Controls *n* (%)	*n* = 1455 AL Cases *n* (%)	Leukemia subtypes	
			*n* = 1253 ALL *n* (%)	*n* = 202 AML *n* (%)
Age Groups (years)				
<5	645 (44.3)	569 (39.1)	515 (41.1)	54 (26.7)
5–10	445 (30.6)	412 (28.1)	358 (28.6)	54 (26.7)
10.1–14	251 (17.3)	282 (19.4)	220 (17.6)	62 (30.7)
>14	114 (7.8)	192 (13.2)	160 (12.8)	32 (15.8)
Birth order				
Firstborn	670 (46.0)	591 (40.6)	507 (40.5)	84 (41.6)
NonFirstborn	785 (54.0)	864 (59.4)	746 (59.5)	118 (58.4)
1–2	1149 (79.0)	1071 (73.6)	925 (73.8)	146 (72.3)
3–4	280 (19.3)	346 (23.8)	296 (23.6)	50 (24.8)
5–6	23 (1.6)	29 (2.0)	26 (2.1)	3 (1.5)
7–8	2 (0.1)	9 (0.6)	6 (0.5)	3 (1.5)
Breastfeeding				
Yes	1272 (87.4)	1330 (91.4)	1152 (91.9)	178 (88.1)
No	183 (12.6)	125 (8.6)	101 (8.1)	24 (11.9)
Breastfeeding (months)				
0–3.9	488 (33.7)	410 (28.3)	354 (28.4)	56 (27.7)
4–6.9	242 (16.7)	281 (19.4)	246 (19.7)	35 (17.3)
7–12.9	467 (32.3)	474 (32.7)	408 (32.7)	66 (32.7)
>13	249 (17.2)	283 (19.5)	238 (19.1)	45 (22.3)
Mother's age (years) at the time of pregnancy				
<20	312 (21.4)	256 (17.6)	210 (16.8)	46 (23.0)
20–24.99	418 (28.7)	477 (32.8)	418 (33.4)	59 (29.5)
25–29.99	375 (25.8)	366 (25.2)	317 (25.3)	49 (24.5)
30–34.99	217 (14.9)	228 (15.7)	195 (15.6)	33 (16.5)
35–39.99	107 (7.4)	109 (7.5)	100 (8.0)	9 (4.5)
≥40	26 (1.8)	17 (1.2)	13 (1.0)	4 (2.0)
Overcrowding				
Yes	864 (59.4)	860 (59.1)	745 (59.5)	115 (56.9)
No	591 (40.6)	595 (40.9)	508 (40.5)	87 (43.1)

AL, acute leukemias; ALL, acute lymphoblastic leukemia; AML, acute myeloid leukemia.

Of the cases, 1253 (86.1%) were ALL and 202 (13.7%) were AML. Of the patients with ALL, 53.3% were male, and with AML, the males were 54.5%. Of the total ALL children, 66.4% were classified as high‐risk patients. A total of 46.1% were diagnosed before the 5 years of age. Table 1 also displays the ORs and 95% CI between birthweight at different categories and AL, ALL, and AML. Using as reference a low birthweight (<2500 g), having a birthweight ≥2500 g was associated with an increased risk of ALL [OR = 2.04 (95% CI: 1.58, 2.63)] and also associated with a higher risk for AML [OR = 1.73 (95% CI: 1.04, 2.87)]. Afterward, a multivariate logistic regression analysis was performed, adjusting by child's sex, birth order, overcrowding index, and age of the mother at the time of pregnancy. We did not include the age of the father for two reasons: (1) because the difference between the incomplete (only considering the age of the mother) model and the complete one (considering both parents′ age) was 0.3127, and (2) we observed a great correlation among the age of both parents. In multivariate analysis, using as reference a birthweight <2500 g, having a birthweight ≥2500 g was associated with an increased risk for the development of ALL [aOR = 2.06 (95% CI: 1.59, 2.66)] (Table 3) and for AML [aOR = 1.77 (95% CI: 1.07, 2.94)]. A birthweight of ≥3500 g was also associated with both leukemia subtypes using as reference a birthweight of <3500 g. Noteworthy, we did not find any association for either of both AL subtypes when a birthweight ≥4000 g was considered.

**Table 3:** Logistic regression analyses between birthweight and risk for the development of acute leukemias in Mexican children

	Acute Leukemias aOR (95% CI)	Acute Lymphoblastic Leukemia aOR (95% CI)	Acute Myeloid Leukemia aOR (95% CI)
Birthweight (in grams)			
≥2500–3499 (*ref.)*	–	–	–
<2500	0.51 (0.39–0.65)	0.49 (0.38–0.64)	0.61 (0.36–1.02)
≥3500	1.10 (0.92–1.30)	1.07 (0.90–1.28)	1.27 (0.91–1.77)
≥4000	1.20 (0.47–3.07)	1.12 (0.41–3.02)	1.95 (0.40–9.40)
<2500 g (*ref.)*	–	–	–
≥2500 g	2.01 (1.58–2.56)	2.06 (1.59–2.66)	1.77 (1.07–2.94)
<3500 g (*ref.)*	–	–	–
≥3500 g	1.22 (1.03–1.43)	1.19 (1.00–1.41)	1.42 (1.03–1.95)
<4000 g (*ref.)*	–	–	–
≥4000 g	1.06 (0.76–1.48)	1.00 (0.70–1.42)	1.43 (0.78–2.59)

aOR, adjusted odds ratio; 95% CI, 95% confidence intervals; *ref.,* reference group. Odds ratios were adjusted for the following variables: child's sex, overcrowding index, birth order, and mother's age at the time of pregnancy.

## Discussion

Our results show a moderate association between relatively appropriate birthweight and ALL as well as for AML. Surprisingly not finding any association with higher birthweight classification (>4000 g), contrary to previous studies reporting an association between high birthweight (>4000 g) and ALL. Hjalgrim 33 in a meta‐analysis of 18 studies pointed out that children weighing ≥4000 g at birth had a greater risk of ALL [OR 1.26, (95% CI: 1.17–1.37)] than children with less weight, and a dose–response effect was also observed (OR 1.14/1000 g increased weight at birth, 95% CI: 1.8–2.06). Nevertheless, a significant association was not evident for AML (OR 1.27, 95% CI: 0.73–2.20). In addition, Caughey 34 in another meta‐analysis of 32 studies adverted an association between a high birthweight and ALL OR 1.23 (95% CI: 1.15, 1.32) and for AML OR 1.40 (95% CI: 1.11, 1.76) when compared to normal birthweight. On the other hand, low birthweight was not associated with ALL, but with AML [OR 1.50 (95% CI: 1.05, 2.13)].

Birthweight directly depends on both intrauterine growth and gestational age. It is of extreme importance to account not only birthweight independently, but at the sight of child's gestational age, in order to establish an association between high birthweight and an accelerated intrauterine growth 44. Milne et al. 45 referred a positive association between the increase of 1 standard deviation in proportion of optimal birthweight (POBW) and risk of ALL (OR 1.18; 95% CI: 1.04, 1.35). Another meta‐analysis of 12 case–control studies of the Childhood Leukemia International Consortium (CLIC) 36 examined the association of ALL using two measures of fetal growth: weight for gestational age and POBW. In summary, the OR was 1.24 (95% CI: 1.13–1.36) for children who were large for gestational age relative to appropriate for gestational age, and the OR was 1.16 (95% CI: 1.09, 1.24) for an increase of 1 standard deviation in POBW. This suggested that an accelerated fetal growth may be associated with an increased risk of ALL in the absence of high birthweight. Other studies have also evidenced the same results 29, 46.

Moreover, as reported in previous studies, an association between accelerated fetal growth and ALL risk is biologically plausible, involving the participation of fetal growth factors, for example, insulin‐like growth factor‐1 (IGF‐1) and IGF‐2, and IGF‐1R receptors, IGF‐2R and its major binding protein‐3 (IGFBP‐3), which have been recognized as crucial in mediating the effect of growth hormones 47, 48, 49. Due to the fact that fetal growth is determined by genetics, nutritional, environmental, and hormonal factors, an exposure to external factors affecting any of these elements could increase the susceptibility to develop genetic alterations, and also alter, levels of IGF‐1, promoting proliferative stress of hematopoietic progenitors, raising the number of cell division and consequently, enhancing the risk of malignant transformation 50, 51, 52, 53, 54.

The present study had a participation rate for leukemia cases of 83.2%. It was not possible to include all the identified cases because we did not have enough matching controls. However, in the Table S1, we attest that there were no differences between cases included in our study and those that were not. Therefore, the possibility of selection bias in the present research was low. Although controls were not interviewed in the same period of recruitment of cases, in the Table S2, we displayed no difference between birthweights′ means and medians among any of controls recruitment years.

Proportion of optimal birthweight has been proposed as the most appropriate measure for assessing the relationship between intrauterine growth velocity and risk of childhood leukemia, as it is dependent of gestational age, and of main nonpathological determinants of intrauterine growth (fetal sex, maternal height, and parity). In the current study, we could not calculate the POBW, for gestational age was not collected, in either cases or controls; instead, we used birthweight which indicates the final fetal weight at the moment of birth. As birthweight was similarly measured in both cases and controls, this could have produced a nondifferential bias with a subestimation of the risk 55.

On the other hand, to control for confounding, we included in multivariate analysis all those variables previously reported as potentially confounders in studies where the relationship between birthweight and AL risk has been investigated 30, 33, 34, 36.

In present study, it was not possible to identify a relationship between weighing at birth ≥4000 g and AL. It could be possibly due to a small number of children presenting this condition. Importantly, when birthweight was categorized as ≥3500 g, a more precise risk estimation was obtained, because sample size and power increased. Therefore, the relationship between AL and a birthweight ≥3500 g could be representing the association between overweight at birth and an increased risk for both types of leukemia.

## Conclusion

In this study, we found a moderate association between not having a low birthweight and an increased risk for both types of leukemia. Birthweight greater than 3500 g was also a risk factor for both types of leukemia. This suggests that a higher birthweight may be associated with an increased risk of developing leukemia in a country with a high incidence of the disease, such as Mexico.

## Conflict of Interest

None declared.

## Supporting information


**Table S1**. Characteristics of leukemia cases registered by MIGICCL (included/not included in present analysis) during the study period (2010–2015).
**Table S2**. Birth weight (in grams) of controls by year of interview.
