# Supplementary material for: A greater birthweight increases the risk of acute leukemias in Mexican children—experience from the Mexican Interinstitutional Group for the Identification of the Causes of Childhood Leukemia (MIGICCL)
Source: Cancer Med. 2018 Mar 13;7(4):1528–36. doi: 10.1002/cam4.1414 (PMC5911591; doi:10.1002/cam4.1414)
Supplement: Supplementary file 1 — Table S1. Characteristics of leukemia cases registered by MIGICCL (included/not included in present analysis) during the study period (2010–2015). Table S2. Birth weight (in grams) of controls by year of interview. [file CAM4-7-1528-s001.rtf]

Supplementary Table 1. Characteristics of leukemia cases registered by MIGICCL (included/not included in present analysis) during study period (2010-2015)	
Characteristics	Total leukemia cases	
	ALL	Other leukemias	Total registered by MIGICCL	
	Included n= 1253 	not included n=228	Included n=202	not included n=70	Included n=1455	not included n=298	
	n(%)	n(%)	n(%)	n(%)	n(%)	n(%)	
Sex	 	 	 	 	 	 	
Male	 	668 (53.3)	124 (54.3)	110(54.5)	41 (58.5)	778(53.5)	165 (55.3)	
Female	 	585(46.7)	104 (45.7)	92(45.5)	29 (41.5)	677(46.5)	133 (44.7)	
Birth weight (in grams)	 	 	 	 	 	
<2500	 	96(7.7)	22 (9.6)	18 (8.9)	2 (2.9)	114 (7.8)	24 (8.1)	
≥2500-3499	 	794(63.4)	153 (67.1)	119(58.9)	49 (70.0)	913(62.7)	202 (67.7)	
≥3500-4500	 	355(28.3	51 (22.3)	63(31.2)	19 (27.1)	418(28.7)	70 (23.4)	
>4500	 	8(0.6)	2 (1.0)	2(1.0)	0	10(0.7)	2 (0.8)	
Birth weight (in grams)	 	 	 	 	 	
<4000	 	1190(95.0)	221 (96.9)	188(93.1)	68 (97.1)	1378(94.7)	289 (96.9)	
>4000	 	63(5.0)	7 (3.1)	14(6.9)	2 (2.9)	77 (5.3)	9 (3.1)	
Birth weight (in grams)	 	 	 	 	 	
<3500	 	890(71.0)	175 (76.7)	136(67.3)	51 (72.8)	1026(70.5)	226 (75.8)	
>3500	 	363(29.0)	53 (23.3)	66(32.7)	19 (27.2)	429 (29.5)	72 (24.2)	
Birth weight (in grams)	 	 	 	 	 	
<2500	 	96(7.7)	22 (9.6)	18(8.9)	2 (2.9)	114(7.8)	24 (8.1)	
>2500	 	1157(92.39	206 (90.4)	184(91.1)	68 (97.1)	1341(92.2)	274 (91.9)	
Age groups (in years)	 	 	 	 	 	 	
<5	 	515(41.1)	86 (37.7)	54 (26.7	20 (28.5)	569(39.1)	106 (35.5)	
5 to 10	 	358(28.6)	58 (25.4)	54(26.7	26 (37.1)	412(28.1)	81 (27.1)	
10.1 to 14	 	220(17.6)	48 (21.1)	62(30.7)	16 (22.8)	282(19.4)	64 (21.5)	
>14	 	160(12.8)	36 (15.8)	32(15.8)	11 (11.6)	192(13.2)	47 (15.9)	
ALL=Acute Lymphoblastic Leukemia, Other leukemias: acute myeloid leukemia, chronic granulocytic leukemia.	


Supplementary Table 2. Birth weight (in grams) of controls by year of interview	
Year of Interview	Total controls (n=1455)	Birth weight (in grams)	
	n	%	median	range (min-max)	p*	mean	SD	p**	
2002	2	0.1	2600	1700-3500	0.1	2600	1272.79	0.09	
2004	52	3.6	3025	1000-4500		3054	686.67		
2005	113	7.8	3150	850-4850		3070	637.45		
2006	22	1.5	3182	1900-4900		3215	633.51		
2007	148	10.2	3187	1250-5300		3133	584.79		
2010	200	13.8	3100	825-4800		3098	664.03		
2011	359	24.7	3000	730-4600		3012	604.64		
2012	418	28.7	3100	900-5200		3121	633.26		
2013	30	2.1	3200	1750-4500		3198	544.58		
2014	13	0.9	2870	1060-3700		2822	713.39		
2015	98	6.7	3035	1000-4500		2955	706.02		
* Kruskall-Wallis ** ANOVA SD=standard deviation	
